# Linear accelerator-based stereotactic radiotherapy for brain metastases, including multiple and large lesions, carries a low incidence of acute toxicities: a retrospective analysis

**DOI:** 10.1186/s13014-023-02262-z

**Published:** 2023-05-10

**Authors:** Toshiki Ikawa, Naoyuki Kanayama, Hideyuki Arita, Shingo Ohira, Koji Takano, Takero Hirata, Masahiro Morimoto, Teruki Teshima, Koji Konishi

**Affiliations:** 1 Department of Radiation Oncology, Osaka International Cancer Institute, Osaka, Japan; 2 Department of Neurosurgery, Osaka International Cancer Institute, Osaka, Japan; 3 Department of Radiation Oncology, Osaka University Graduate School of Medicine, Osaka, Japan; 4 Osaka Heavy Ion Therapy Center, Osaka, Japan

**Keywords:** Brain neoplasms, Radiosurgery, Toxicity, Linear accelerator

## Abstract

**Background:**

Data on acute toxicities after stereotactic radiotherapy (SRT) for brain metastases, including multiple and large lesions, are lacking. We aimed to evaluate the incidence and nature of toxicities immediately after SRT using a linear accelerator.

**Methods:**

This retrospective study reviewed the medical records of 315 patients with brain metastases treated with SRT at our institution between May 2019 and February 2022. In total, 439 SRT sessions were performed for 2161 brain metastases. The outcome of interest was immediate side effects (ISEs), defined as new or worsening symptoms occurring during SRT or within 14 days after the end of SRT.

**Results:**

Grade ≥ 2 and ≥ 3 ISEs occurred in 16 (3.6%) and 7 (1.6%) cases, respectively. Among 63 treatments for 10 or more lesions (range: 10–40), 1 (1.6%) ISE occurred. Among 22 treatments for lesions with a maximum tumor volume of > 10 cc, 2 (9.1%) ISEs occurred. Grade ≥ 3 ISEs included 1, 4, 1, and 1 cases of grade 3 nausea, grade 3 new-onset partial and generalized seizures, grade 3 obstructive hydrocephalus, and grade 5 intracranial hemorrhage, respectively. ISEs were more common in patients with a larger maximum tumor volume, primary sites other than lung and breast cancer, and pre-treatment neurological symptoms.

**Conclusion:**

SRT using a linear accelerator for brain metastases, including multiple and large lesions, is safe, with a low incidence of ISEs. Serious complications immediately after SRT are rare but possible; therefore, careful follow-up is necessary after treatment initiation.

**Supplementary Information:**

The online version contains supplementary material available at 10.1186/s13014-023-02262-z.

## Background

Stereotactic radiotherapy (SRT) is an essential treatment modality for brain metastases [1, 2]. Brain injury after radiotherapy has customarily been categorized into acute, early delayed, and late reactions. In general, acute reactions occur within days to weeks after treatment initiation, whereas early delayed reactions occur from 1 to 6 months, and late reactions occur from 6 months [3]. The primary concern for SRT toxicity is brain necrosis, which occurs as a late reaction, and many studies have investigated its characteristics and dose-volume effects [4–7]. In addition, SRT can cause new or worsening symptoms immediately after treatment initiation. These acute reactions include nausea/vomiting, dizziness, headache, seizures, and neurological deficits, and they can be severe [8]. However, these toxicities have been less studied, and their actual risk remains poorly understood [9]. Recent advances in SRT with linear accelerators have made it possible to treat multiple brain metastases simultaneously [10, 11]. Multi-fraction SRT has been increasingly used to treat larger brain metastases [7, 12]. Treating multiple lesions simultaneously or treating large lesions increases the dose to the normal brain and might increase these toxicities. This study aimed to evaluate the incidence and nature of acute toxicities immediately after SRT for brain metastases, including multiple and large lesions, using linear accelerators.

## Methods

### Study design and patients

This retrospective study was approved by the ethics committee of Osaka International Cancer Institute (approval number 21150) and was conducted according to the tenets of the Declaration of Helsinki. All patients provided written informed consent for using their data for clinical research before the administration of radiotherapy and had the opportunity to opt out of the study. From our electronic database, we identified 511 consecutive SRT treatments of brain metastases performed at our institution between May 2019 and February 2022. Among these, treatments for postoperative cavities or recurrence (n = 32), for meningeal metastases (n = 12), for more than 40 metastases (n = 2), with no adverse events but less than 14 days of follow-up after the end of SRT (n = 18), not completed for reasons other than adverse events (n = 4), and combining single and multi-fraction SRT (n = 4) were excluded. In total, 315 patients, 439 treatments, and 2161 metastases were included in the study.

### SRT protocol

SRT treatment was performed as described previously [13–15]. All patients were immobilized using a thermoplastic mask, and planning computed tomography was performed using an iodine contrast agent, unless medically contraindicated. The gross tumor volume (GTV) was delineated using T1-weighted gadolinium-enhanced magnetic resonance images. When a gadolinium contrast agent was contraindicated, contrast-enhanced planning computed tomography images or T2-weighted magnetic resonance images were used. The planning target volume (PTV) was generated by adding an isotropic margin of 1 mm to the GTV. Increasing or decreasing the margin from 0–3 mm was allowed based on the patient’s condition.

We ordinarily prescribed 20–24 Gy in one fraction for GTV < 4 cc and 30 Gy in three fractions or 30–35 Gy in five fractions for GTV > 4 cc. Since 2020, 35 Gy in five fractions was generally prescribed in all cases. The dose was prescribed to cover 95% or 99% of combined PTVs. All treatments were performed using automated non-coplanar volumetric-modulated arc therapy (HyperArc; Varian Medical Systems, Palo Alto, CA) or non-coplanar dynamic conformal arc therapy with a linear accelerator equipped with a 2.5-mm multileaf collimator (TrueBeam STx or Edge; Varian Medical Systems).

Corticosteroids were administered when neurological symptoms were present or when peritumoral brain edema was strong, and there was a risk of symptom emergence. Anticonvulsants were not administered prophylactically. Typically, betamethasone was administered at a dose of 1–2 mg/day and 3–16 mg/day for prophylactic and therapeutic use, respectively, to treat brain edema and then tapered off during the first 1–3 weeks after SRT.

### Outcome evaluation

Based on studies by Werner-Wasik et al. and George et al., immediate side effects (ISEs) were defined as new or unexpected symptoms occurring during SRT or within 14 days after the end of SRT [8, 16]. These included cases of unexpected worsening of neurological symptoms before treatment. ISE grading was according to the Common Terminology Criteria for Adverse Events v5.0. ISEs included neurological and non-neurological adverse events related to SRT, such as cerebral hemorrhage and hydrocephalus. Mild headache and nausea (grade 1) were not considered ISEs. When cerebral edema occurred or worsened, the associated neurological symptoms were considered adverse events; however, the occurrence or worsening of cerebral edema alone was not considered an adverse event.

### Statistical analyses

To examine the association between ISEs and patient, tumor, and treatment characteristics, statistical analyses were performed in patients who did and did not develop ISEs. Given that some patients underwent more than one session of SRT, statistical analyses were performed based on treatment. Spearman’s correlation coefficient was used to determine the correlation between variables. Differences in baseline characteristics between the two groups were assessed using the Wilcoxon rank-sum test for continuous variables and the Fisher’s exact test or chi-square test for categorical variables. Multiple testing adjustments were not used because of the exploratory nature of the study. All analyses were performed using R software (version 4.1.1) (R Foundation for Statistical Computing, Vienna, Austria). All statistical tests were two-sided, and p < 0.05 was considered statistically significant.

## Results

### Patient, tumor, and treatment characteristics

Of the 439 treatment courses, 94 (21%) courses were performed using 1-fraction SRT, 249 (57%) courses with 5-fraction SRT, and 96 (22%) courses with 2 or 3–10 fractions of SRT. The patient, tumor, and treatment characteristics are shown in Table 1. The median number of brain metastases treated simultaneously per treatment was 2 (range: 1–40). The median maximum tumor volume per treatment was 0.62 cc (interquartile range [IQR]: 0.17–2.50 cc; range: 0.01–33.25 cc), and the median value of the total tumor volume per treatment was 1.07 cc (IQR: 0.31–3.63 cc; range: 0.01–66.58 cc). The median isodose (prescription dose/max dose × 100) per treatment was 53.3% (IQR: 49.8–69.3%; range: 37.8–95.8%). The distributions of the dose fractionation, isodose, number of metastases, maximum tumor volume, total tumor volume, and primary tumor are shown in Additional file 1. The most common histology was non-small cell lung cancer (53%), followed by small-cell lung cancer (14%) and breast cancer (13%). In 31 (7.1%) treatments, patients had previously received whole-brain irradiation. There were 24 (5.5%) treatments performed for lesions previously treated with SRT. In 80 (18%) treatments, patients had neurological symptoms before treatment. In 136 (31%) treatments, the patients received corticosteroids during the SRT treatment period. In 178 (40%), 37 (8.4%), 103 (24%), and 62 (14%) treatments, the patients received cytotoxic chemotherapeutic agents, molecularly targeted agents with anti-vascular endothelial growth factor (VEGF) activity, other molecularly targeted agents, and immune checkpoint inhibitors, respectively, within 30 days before SRT or during SRT.

**Table 1:** Patient, tumor, and treatment characteristics

Characteristic	N = 439
Age, years	67 (55, 74) [25–88]
Sex	
Female	213 (48.5%)
Male	226 (51.5%)
Prescribed dose/Number of fractions	
20–24 Gy in 1 fraction	94 (21.4%)
30 Gy in 3 fractions	72 (16.4%)
30–35 Gy in 5 fractions	249 (56.7%)
40–42 Gy in 10 fractions	10 (2.3%)
Others	14 (3.2%)
Prescribed dose/Max dose, %	53.3 (49.8, 69.3) [37.8–95.8]
Number of metastases	
1	159 (36.2%)
2–4	143 (32.6%)
5–9	74 (16.9%)
10–19	43 (9.8%)
20–40	20 (4.6%)
Maximum tumor volume, cc	0.62 (0.17, 2.50) [0.01–33.25]
Total tumor volume, cc	1.07 (0.31, 3.63) [0.01–66.58]
Primary cancer	
Lung, non-small cell	232 (52.8%)
Lung, small cell	61 (13.9%)
Breast	57 (13.0%)
Gastrointestinal tract	37 (8.4%)
Kidney	12 (2.7%)
Melanoma	17 (3.9%)
Others	23 (5.2%)
Prior history of whole-brain radiotherapy	31 (7.1%)
Re-irradiation of lesions previously treated with SRT	24 (5.5%)
Presence of neurological signs	80 (18.2%)
Use of corticosteroids	136 (31.0%)
Receipt of cytotoxic agents	178 (40.5%)
Receipt of molecularly targeted agents with anti-VEGF activity	37 (8.4%)
Receipt of other molecularly targeted agents	103 (23.5%)
Receipt of immune checkpoint inhibitors	62 (14.1%)

Data are presented as the median (interquartile range) [minimum–maximum] or as n (%). SRT, stereotactic radiotherapy; VEGF, vascular endothelial growth factor

### ISEs

ISEs occurred in 16 (3.6%) treatments within a median of 5 days (range: 0–14 days) after SRT initiation. ISEs are summarized in Table 2, and the characteristics of all patients who experienced ISEs are described in Additional file 2. Grade ≥ 2 and ≥ 3 ISEs were observed in 16 (3.6%) and 7 (1.6%) cases, respectively. Among the 63 treatments for 10 or more lesions, 1 (1.6%) ISE occurred. The incidence of ISEs was 2 (9.1%) among the 22 treatments for lesions with a maximum tumor volume > 10 cc. Grade 3 new-onset seizures occurred in 4 (0.9%) patients; 2 of these patients had partial seizures and 2 had complex partial or generalized seizures. They were treated with corticosteroids or anticonvulsants and their symptoms improved. Grade ≥ 3 ISEs other than seizures included 1 case of grade 3 obstructive hydrocephalus, 1 case of grade 3 nausea, and 1 case of grade 5 intracranial hemorrhages. The first patient, who had a single large cerebellar metastasis of 25.9 cc from ovarian cancer, presented with severe headache and nausea after SRT initiation and was diagnosed with obstructive hydrocephalus using computed tomography. Her symptoms improved without surgical intervention after administration of corticosteroids and osmotic diuretics. The second patient, who had two metastases from lung cancer, including a cerebellar metastasis, presented with grade 3 nausea after SRT initiation. Computed tomography showed worsening of peritumoral edema in the cerebellum. The patient received an increased dose of corticosteroids, but the symptoms improved slowly. The last patient, who had multiple metastases from melanoma, developed multiple grade 5 hemorrhages after the end of SRT.

**Table 2:** Summary of immediate side effects

Grade	n (%)
Grade 2	
Nausea	3 (0.7%)
Cognitive disturbance	1 (0.2%)
Ataxia	1 (0.2%)
Dysesthesia	1 (0.2%)
Dysphasia	1 (0.2%)
Muscle weakness	4 (0.9%)
Nervous system disorders—Other (visual field defect)	1 (0.2%)
Grade 3	
Headache	1 (0.2%)
Nausea	2 (0.5%)
Seizure (partial)	2 (0.5%)
Seizure (complex partial or generalized)	2 (0.5%)
Hydrocephalus	1 (0.2%)
Grade 5	
Intracranial hemorrhage	1 (0.2%)

**Fig. 1:**
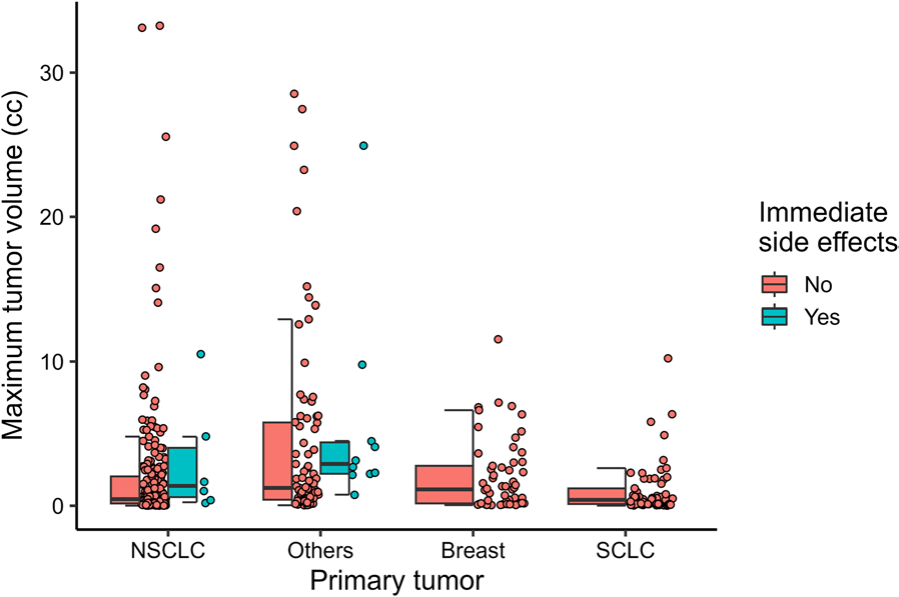
Dot plots and boxplots of maximum tumor volume in the treatments for non-small-cell lung cancer (NSCLC), others, breast cancer, and small-cell lung cancer (SCLC) primary site. Dots, maximum tumor volume; boxes, median tumor volume, and upper/lower quartiles; whiskers, maximum and minimum tumor volume within 1.5 × interquartile range

Patient characteristics were compared between the ISE and no-ISE groups (Table 3). The distributions of the dose fractionation, isodose, number of metastases, maximum tumor volume, total tumor volume, and primary tumor, stratified by the occurrence of ISE, are shown in Additional file 1. The total tumor volume was excluded from the analysis because it was strongly correlated with the maximum tumor volume (r_s_ = 0.95). The ISE group had a significantly larger maximum tumor volume (median: 2.49 vs. 0.61 cc; p = 0.001). The incidence of ISE differed according to the primary site (p < 0.001). It was significantly higher in primary sites other than lung and breast cancer (pairwise comparisons are shown in Additional file 3). Patients with neurological symptoms before treatment had a higher incidence of ISEs than those without neurological symptoms (p < 0.001). The relationship between the maximum tumor volume, primary site, and ISEs is shown in Fig. 1. No ISEs occurred in breast or small-cell lung cancer, although some cases had a maximum tumor volume similar to that in cases of ISEs in non-small-cell lung cancer and other primary sites. The relationship between corticosteroid use and the incidence of ISEs stratified by pre-treatment neurological symptoms is shown in Table 4. In patients with pre-treatment neurological symptoms, the incidence of ISEs was significantly lower in patients who received corticosteroids than in those who did not (6.2% and 31.2%, respectively; p = 0.014). In the group of patients without pre-treatment neurological symptoms, the difference in ISE incidence between patients who received corticosteroids and those who did not was small (0% and 2.4%, respectively; p = 0.4).

**Table 3:** Comparison of characteristics between patients who did and did not develop immediate side effects

Characteristic	Immediate side effects		
	No, N = 423^1^	Yes, N = 16^1^	p-value^2^
Age, years	67 (56, 74) [25–88]	60 (41, 70) [30–77]	0.050
Sex			0.37
Female	207 (48.9%)	6 (37.5%)	
Male	216 (51.1%)	10 (62.5%)	
Prescribed dose/Number of fractions			0.27
20–24 Gy in 1 fraction	93 (22.0%)	1 (6.2%)	
30 Gy in 3 fractions	68 (16.1%)	4 (25.0%)	
30–35 Gy in 5 fractions	239 (56.5%)	10 (62.5%)	
40–42 Gy in 10 fractions	9 (2.1%)	1 (6.2%)	
Others	14 (3.3%)	0 (0.0%)	
Prescribed dose/Max dose, %	53.3 (49.8, 69.9) [37.8–95.8]	51.7 (50.3, 59.4) [47.8–81.6]	0.70
Number of metastases			0.56
1	154 (36.4%)	5 (31.2%)	
2–4	135 (31.9%)	8 (50.0%)	
5–9	72 (17.0%)	2 (12.5%)	
10–40	62 (14.7%)	1 (6.2%)	
Maximum tumor volume, cc	0.61 (0.17, 2.42) [0.01–33.25]	2.49 (1.54, 4.56) [0.24–24.92]	0.001
Primary cancer			< 0.001
Lung, non-small cell	226 (53.4%)	6 (37.5%)	
Lung, small cell	61 (14.4%)	0 (0.0%)	
Breast	57 (13.5%)	0 (0.0%)	
Others	79 (18.7%)	10 (62.5%)	
Prior history of whole-brain radiotherapy	31 (7.3%)	0 (0.0%)	0.62
Re-irradiation of lesions previously treated with SRT	24 (5.7%)	0 (0.0%)	> 0.99
Presence of neurological signs	71 (16.8%)	9 (56.2%)	< 0.001
Use of corticosteroids	132 (31.2%)	4 (25.0%)	0.78
Receipt of cytotoxic agents	174 (41.1%)	4 (25.0%)	0.20
Receipt of molecularly targeted agents with anti-VEGF activity	36 (8.5%)	1 (6.2%)	> 0.99
Receipt of other molecularly targeted agents	101 (23.9%)	2 (12.5%)	0.38
Receipt of immune checkpoint inhibitors	57 (13.5%)	5 (31.2%)	0.060

^a^Data are presented as the median (interquartile range) [minimum–maximum] or as n (%)

^b^Wilcoxon rank-sum test; Pearson’s chi-squared test; Fisher’s exact test. SRT, stereotactic radiotherapy; VEGF, vascular endothelial growth factor

**Table 4:** Relationship between corticosteroid use and incidence of immediate side effects stratified by the presence of neurological signs

Use of corticosteroids	Presence of neurological signs							
	No				Yes			
	No	Yes	Total	p-value^a^	No	Yes	Total	p-value^a^
Incidence of immediate side effects				0.35				0.014
No	280 (97.6%)	72 (100%)	352 (98.1%)		11 (68.8%)	60 (93.8%)	71 (88.8%)	
Yes	7 (2.4%)	0 (0%)	7 (1.9%)		5 (31.2%)	4 (6.2%)	9 (11.2%)	

^a^Fisher’s exact test

## Discussion

The actual risk of acute toxicities after SRT for brain metastases is poorly understood. The reported incidence of acute toxicities in SRT for brain metastases varies among studies, ranging from 0–25.8% [17–27]. This may be due to the heterogeneous patient backgrounds, different definitions of outcomes, and different observation periods. This study defined toxicity within the first 2 weeks after treatment initiation as an ISE. The incidence rate (3.6%) of ISEs was low, and the incidence of grade ≥ 3 ISE was 1.6%. The current study included patients with 10 or more lesions and large lesions (> 10 cc); however, the incidence of ISEs for these lesions was acceptable at 1.6% and 9.1%, respectively. Given that the number of ISEs did not increase as the number of tumors increased, SRT with the current linear accelerator, which can treat many tumors simultaneously in a single isocenter, is considered safe for brain metastases.

In the current study, the incidence of ISEs was associated with maximum tumor volume, primary site, and pre-treatment neurological symptoms. This finding was consistent with those of Lerner et al. [28] who reported that total tumor volume and pre-SRT neurological symptoms were associated with the development of seizures after SRT. However, one patient in the current study had a generalized seizure, despite having a single and relatively small lesion (0.24 cc). Seizures can lead to injury or accidents [29]; thus, it is crucial to explain the possibility of seizures in patients undergoing SRT before initiating treatment, even for small lesions.

Interestingly, this study showed no adverse events in breast or small-cell lung cancer. In contrast, significantly more adverse events occurred in other primary sites, suggesting that the incidence of ISEs varies by histological type. The development of neurological symptoms is related to not only the mass effect of the tumor but also various factors, including peritumoral vasogenic edema [9, 30, 31]. For example, vasogenic edema is thought to be caused by the breakdown of the blood–brain barrier and the subsequent increase in interstitial fluid. Vasogenic edema is mediated by molecular factors such as VEGF [31, 32]. Irradiation to tumors may influence these molecular expressions and enhance peritumoral edema [33]; thus, the occurrence of acute toxicities after SRT may differ by histological type and their molecular expressions. Indeed, Hanna et al. [34] compared pre- and post-SRT magnetic resonance images and found that edema exacerbation varies by histological type, with renal cell carcinoma and melanoma being more prone to edema exacerbation than other histological types. Molecularly targeted drugs, especially those with anti-VEGF activity, might alter the incidence of toxicities. Although no association between these drugs and ISEs was found in this study, further investigation is warranted because multivariable analyses were not performed due to the low incidence of ISEs, which is a limitation of this study.

We observed that corticosteroid administration reduced the incidence of ISEs in patients with pre-treatment neurological symptoms. The use of corticosteroids for symptoms caused by brain tumors is widely accepted [31, 35]. However, physicians may avoid administering corticosteroids to patients scheduled to receive immunotherapy even if they are symptomatic because of concerns that corticosteroids may decrease the effectiveness of immunotherapy [36]. In the current study, worsening of neurological symptoms occurred in 31% of patients who had pre-treatment neurological symptoms but did not receive corticosteroids. Such patients are considered at high risk for acute toxicities and require caution. In the group without pre-treatment neurological symptoms, the difference in ISE incidence was small between patients who received corticosteroids and did not. We have ordinarily administered prophylactic corticosteroids to patients with severe peritumoral edema, suggesting that prophylactic administration of corticosteroids may reduce the incidence of acute toxicities in asymptomatic patients with severe peritumoral edema. There is no consensus on the use of prophylactic administration of corticosteroids [9], and further studies considering peritumoral edema are needed to identify groups that would benefit from prophylactic administration of corticosteroids.

In the current study, there were no significant differences in the occurrence of symptoms according to dose fractionation. Patients with large lesions and those who had pre-treatment neurological symptoms were more often treated with multi-fraction SRT, and the present analysis could not conclude whether this factor influenced the occurrence of ISE. Although a few studies reported that acute toxicities were less common in multi-fraction SRT than in single-fraction SRT [20, 27, 37], the effects of multi-fraction SRT on acute toxicities require further investigation.

Among the three cases of grade ≥ 3 toxicities excluding seizures, two patients had metastasis in the posterior cranial fossa. Obstructive hydrocephalus occurred in one case, and drug-refractory nausea occurred in one case. One hydrocephalus case had an extensive 25.9-cc metastasis in the cerebellum, and the cerebral fluid pathway was compressed. One drug-refractory nausea case had a 4.8-cc metastasis in the cerebellum. Posterior cranial fossa lesions are generally considered more symptomatic due to the small space of the posterior cranial fossa and the risk of obstructing the cerebral fluid pathways and causing hydrocephalus [31, 38]. Given that SRT-induced edema could cause severe toxicities in patients with large lesions or lesions located near the cerebral fluid pathways in the posterior cranial fossa, we believe that there should be a discussion with the neurosurgical oncologist regarding whether such lesions should be treated with surgery or SRT and how to respond to toxicities when they occur. Grade 5 intracranial hemorrhage occurred in one case. Yomo et al. [39] reported a tumor hemorrhage incidence of 0.33% in 905 SRT cases, similar to ours. Although rare, clinicians should be aware of the possibility of severe hemorrhage during and after treatment. Melanomas are prone to hemorrhage and may require special attention [22, 40]. Liew et al. [40] reported that tumor hemorrhage occurred in 64 (25%) of 259 patients with melanoma brain metastases within a median time of 1.6 months after SRT.

This study has some limitations. As this was a single-center analysis, multicenter studies are needed to confirm the external validity of our findings. The presence of toxicities was retrospectively determined from medical records, which may have introduced observer bias. It was also difficult to distinguish between SRT toxicities and worsening symptoms during the natural course of the disease due to tumor progression. The incidence of toxicities was low, and multivariate analysis was not performed. Parameters related to brain dose were not analyzed in this study because they were observed to correlate with maximum tumor volume and total tumor volume. The relationship between specific tumor locations and the occurrence of toxicities was not investigated owing to the inclusion of patients with multiple metastases. Further studies are needed to establish predictors of acute toxicities, such as enrolling more patients or considering tumor location.

## Conclusions

In conclusion, SRT with a linear accelerator for brain metastases is safe, with a low incidence of acute toxicities. Serious complications are rare but possible; therefore, careful follow-up is necessary during and after treatment.

## Supplementary Information

**Additional file 1: Figures S1–S6**. Stacked histogram and bar chart of dose fractionation, isodose, number of metastases, maximum tumor volume, total tumor volume, and primary tumor, stratified by the occurrence of immediate side effects.

**Additional file 2: Table S1**. Characteristics of all patients who experienced immediate side effects.

**Additional file 3: Table S2**. Pairwise comparisons of the incidence of immediate side effects, stratified by primary sites.

## Data Availability

The datasets generated during and/or analyzed during the current study are available from the corresponding author on reasonable request.

## Abbreviations

SRT: Stereotactic radiotherapy
GTV: Gross tumor volume
ISE: Immediate side effects
IQR: Interquartile range
NSCLC: Non-small-cell lung cancer
PTV: Planning target volume
SCLC: Small-cell lung cancer
VEGF: Vascular endothelial growth factor
