# Supplementary material for: Linear accelerator-based stereotactic radiotherapy for brain metastases, including multiple and large lesions, carries a low incidence of acute toxicities: a retrospective analysis
Source: Radiat Oncol. 2023 May 10;18:80. doi: 10.1186/s13014-023-02262-z (PMC10173492; doi:10.1186/s13014-023-02262-z)
Supplement: Supplementary file 1 — Additional file 1: Figures S1–S6. Stacked histogram and bar chart of dose fractionation, isodose, number of metastases, maximum tumor volume, total tumor volume, and primary tumor, stratified by the occurrence of immediate side effects. [file 13014_2023_2262_MOESM1_ESM.docx]

**Additional Figures 1**–**6**

**
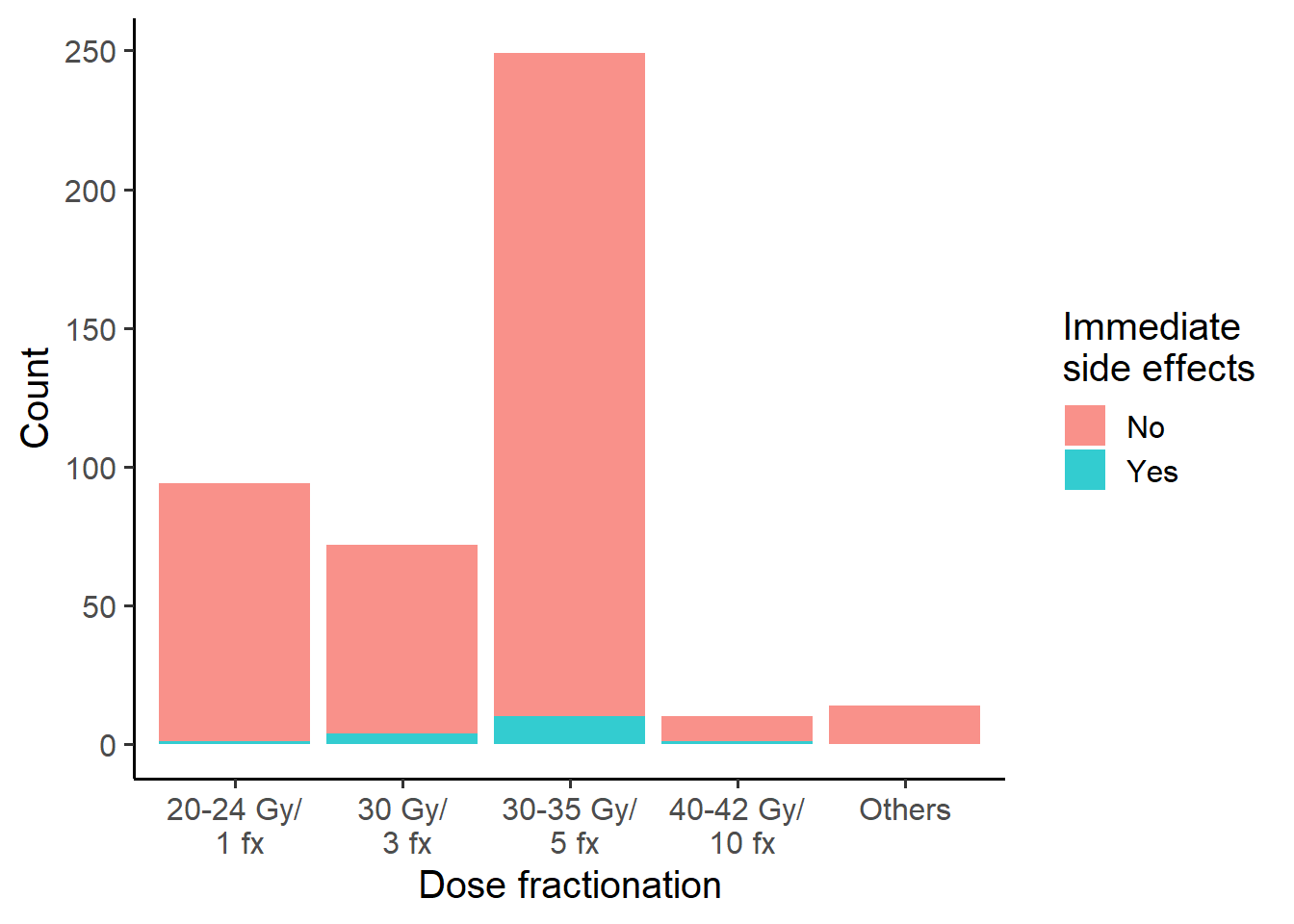
**

**Additional** **Figure 1.** Stacked bar chart of the dose fractionation stratified by occurrence of immediate side effects.


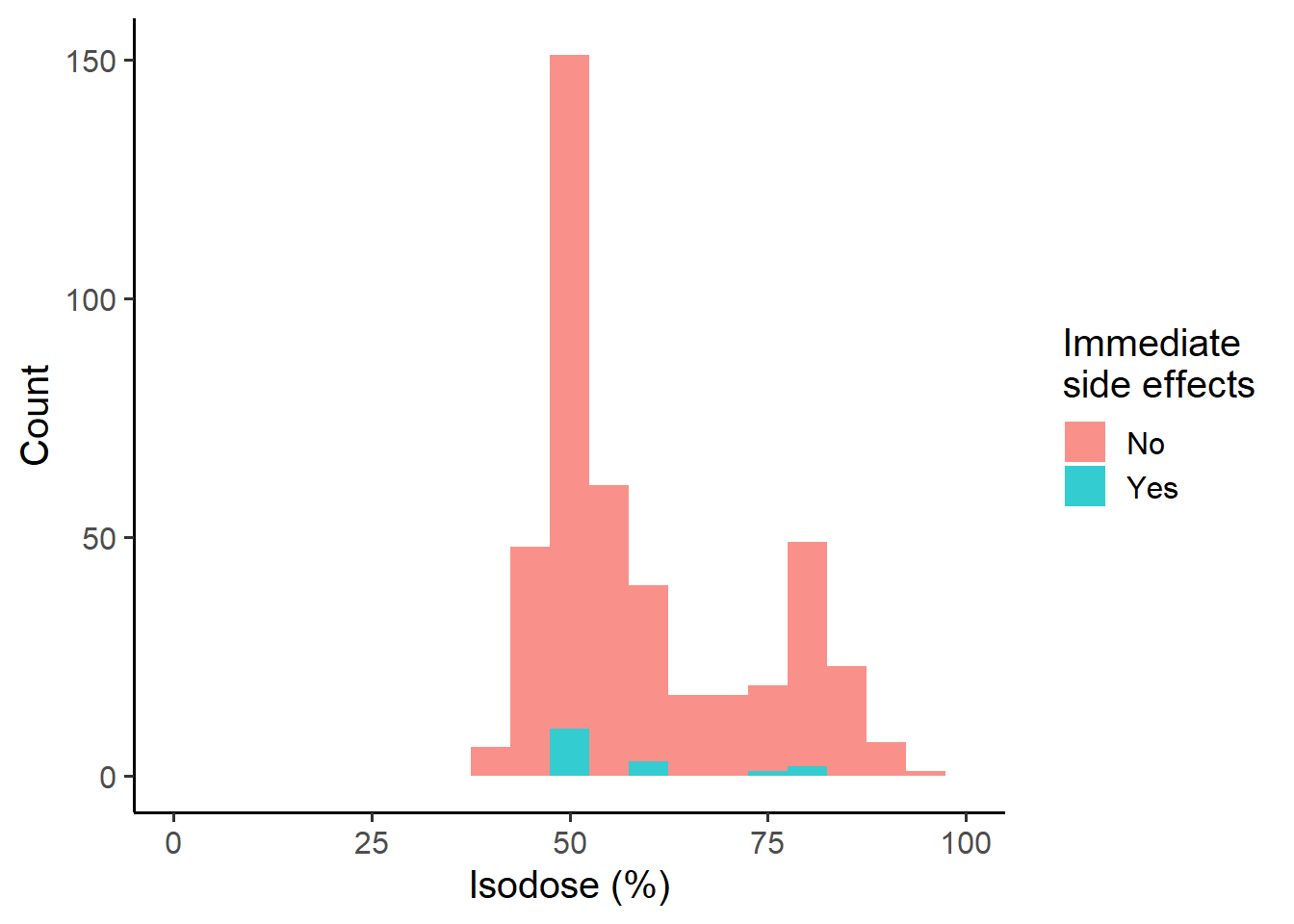


**Additional** **Figure** **2.** Stacked histogram of the isodose (prescription dose/max dose × 100) stratified by occurrence of immediate side effects.

**
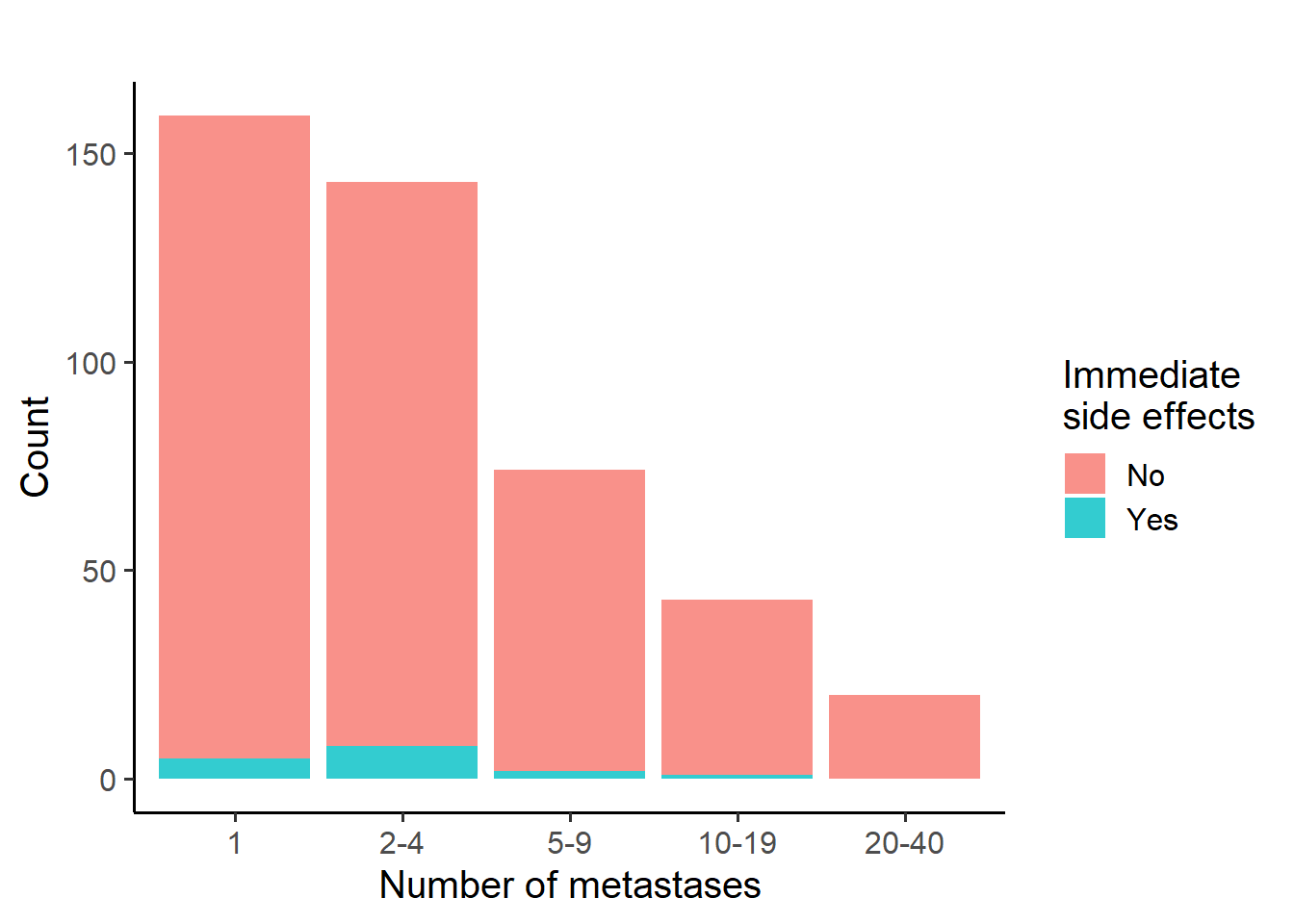
**

**Additional** **Figure** **3.** Stacked bar chart of the number of metastases stratified by occurrence of immediate side effects.

**
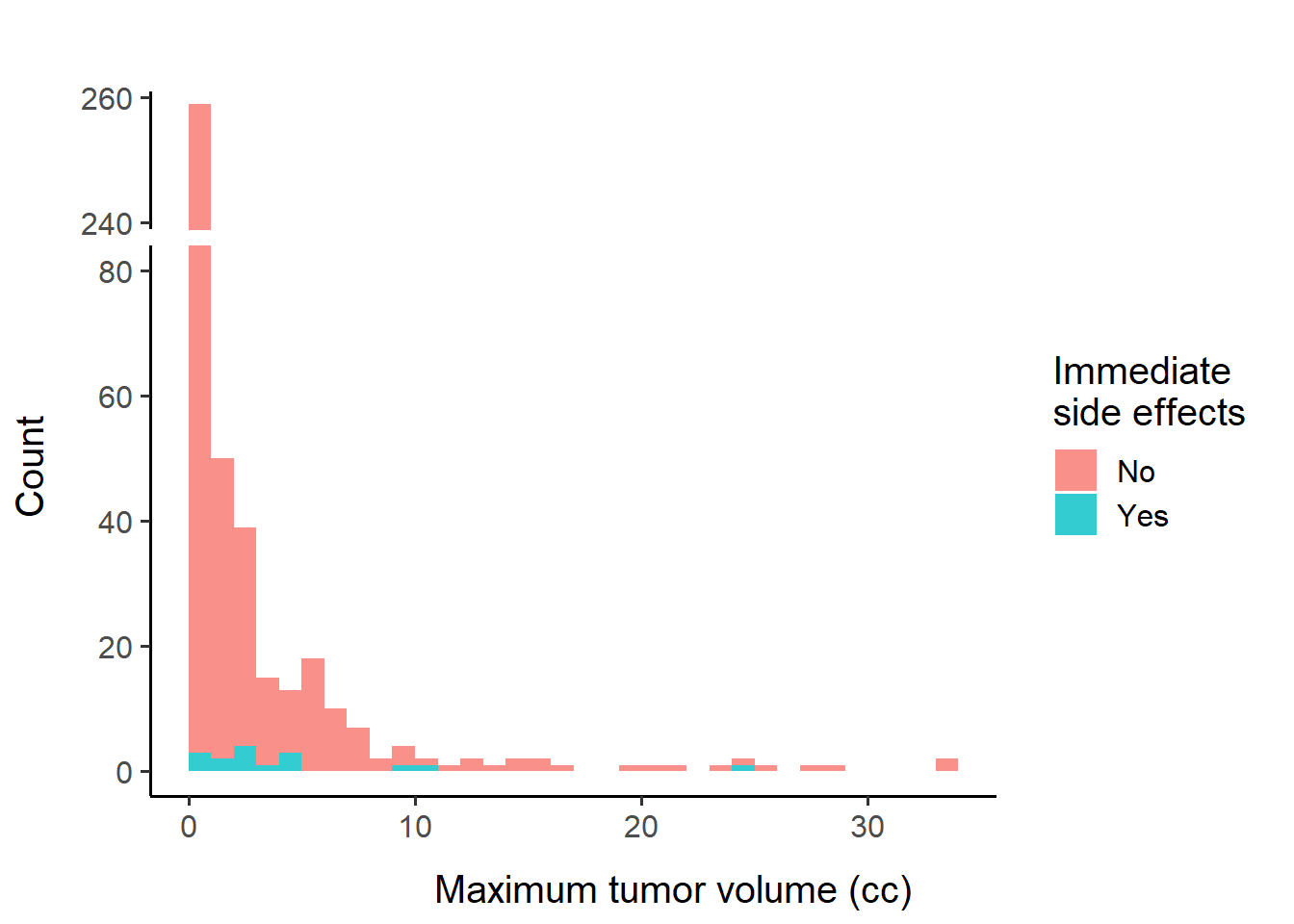
Additional** **Figure** **4.** Stacked histogram of the maximum tumor volume stratified by occurrence of immediate side effects. The break in the Y-axis was introduced using the ggbreak package in R [1].

**
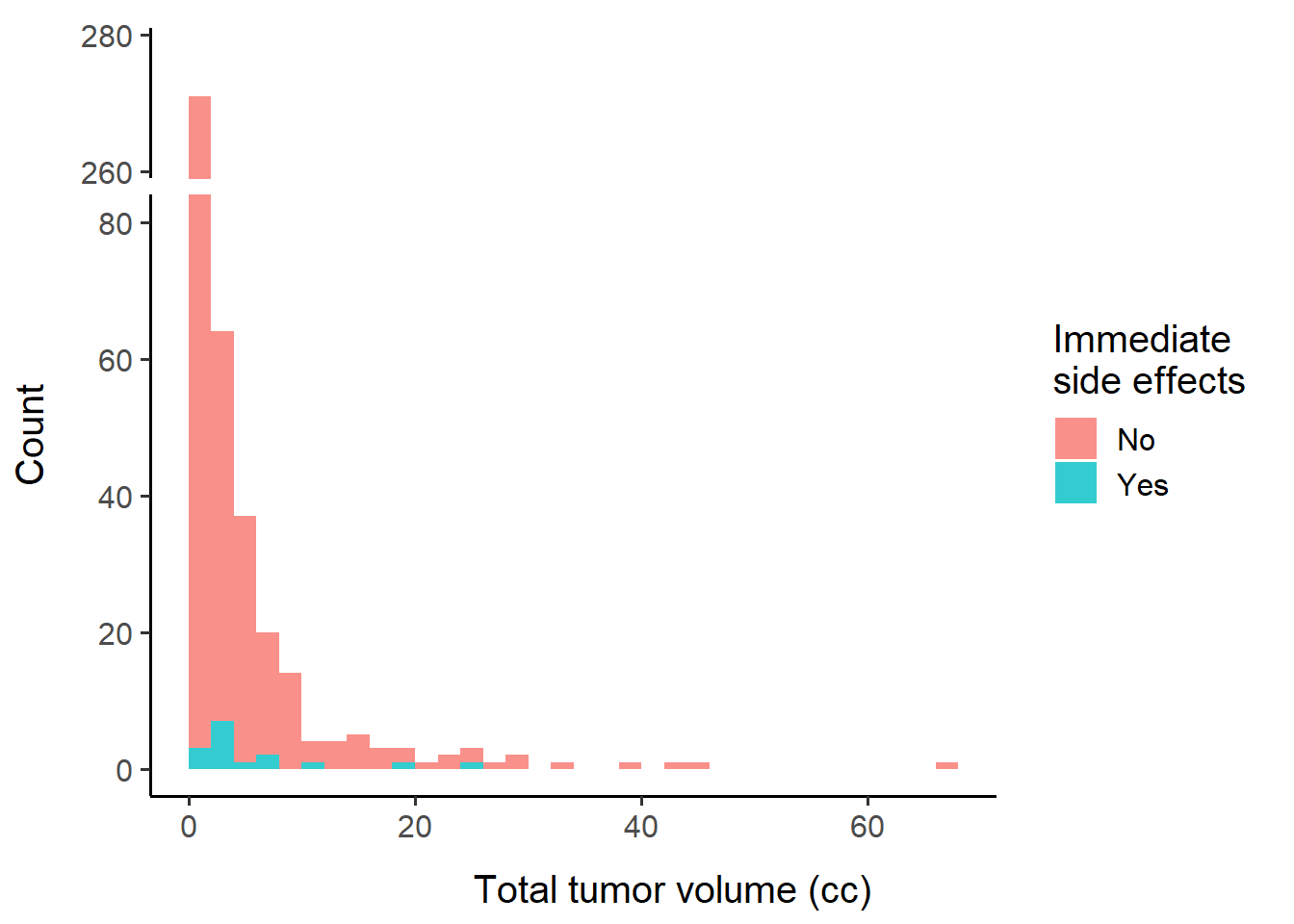
**

**Additional** **Figure** **5.** Stacked histogram of the total tumor volume stratified by occurrence of immediate side effects. The break in the Y-axis was introduced using the ggbreak package in R [1].

**
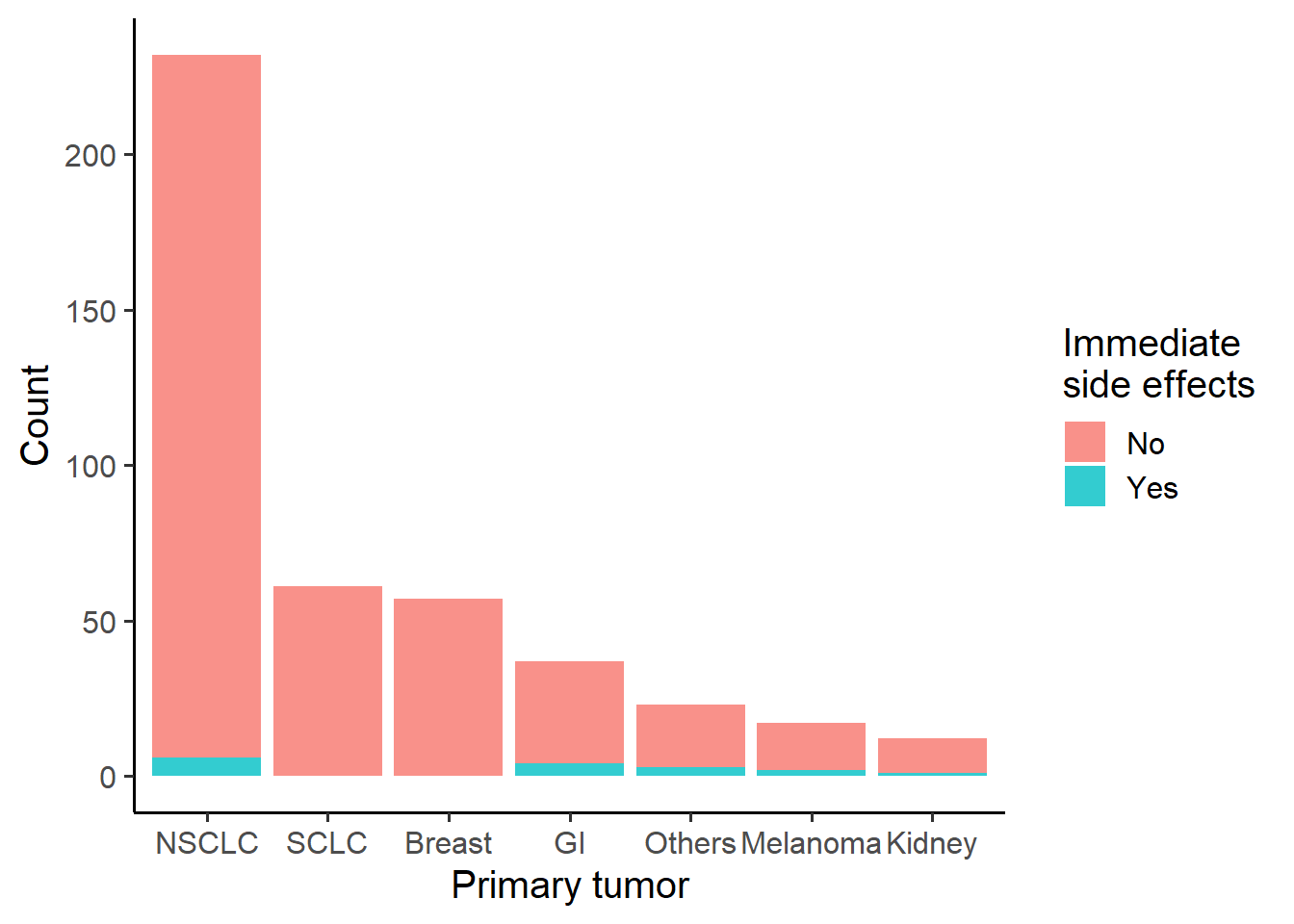
**

**Additional** **Figure** **6.** Stacked bar chart of the primary tumor stratified by occurrence of immediate side effects.

**Reference for** **Additional** **Figures** **4 and 5**

1. Xu S, Chen M, Feng T, Zhan L, Zhou L, Yu G. Use *ggbreak* to effectively utilize plotting space to deal with large datasets and outliers. Front Genet. 2021;12:774846. doi: 10.3389/fgene.2021.774846
