# Supplementary material for: Linear accelerator-based stereotactic radiotherapy for brain metastases, including multiple and large lesions, carries a low incidence of acute toxicities: a retrospective analysis
Source: Radiat Oncol. 2023 May 10;18:80. doi: 10.1186/s13014-023-02262-z (PMC10173492; doi:10.1186/s13014-023-02262-z)
Supplement: Supplementary file 3 — Additional file 3: Table S2. Pairwise comparisons of the incidence of immediate side effects, stratified by primary sites. [file 13014_2023_2262_MOESM3_ESM.docx]

**Additional Table 2.** Pairwise comparisons of the incidence of immediate side effects, stratified by primary sites

| Group 1 | Group 2 | p-value |
| --- | --- | --- |
| Breast | NSCLC | 0.60 |
| Breast | SCLC | 1 |
| NSCLC | SCLC | 0.35 |
| Others | NSCLC | 0.0030 |
| Others | Breast | 0.0067 |
| Others | SCLC | 0.0057 |

p-values were calculated using Fisher’s exact test or chi-square test without multiple testing adjustments. Abbreviations: NSCLC, non-small-cell lung cancer; SCLC, small-cell lung cancer
